# Low Serum ZAG Levels Correlate With Determinants of the Metabolic Syndrome in Chinese Subjects

**DOI:** 10.3389/fendo.2020.00154

**Published:** 2020-03-24

**Authors:** Linjie Wang, Meijuan Liu, Dongping Ning, Huijuan Zhu, Guangliang Shan, Dingming Wang, Bo Ping, Yangwen Yu, Hongbo Yang, Kemin Yan, Hui Pan, Fengying Gong

**Affiliations:** ^1^Key Laboratory of Endocrinology of National Health Commission, Department of Endocrinology, Peking Union Medical College Hospital, Chinese Academy of Medical Science and Peking Union Medical College, Beijing, China; ^2^Department of Epidemiology and Health Statistics, Institute of Basic Medical Sciences, Peking Union Medical College Hospital, Chinese Academy of Medical Science and Peking Union Medical College, Beijing, China; ^3^Guizhou Provincial Center for Disease Control and Prevention, Guiyang, China; ^4^Longli Center for Disease Control and Prevention, Longli, China

**Keywords:** zinc-α2-glycoprotein (ZAG), metabolic syndrome (MetS), central obesity, international diabetes federation (IDF), biomarker

## Abstract

**Introduction:** Zinc-α2-glycoprotein (ZAG) is a novel adipokine, which is involved in metabolic syndrome (MetS). This study aimed to investigate the relationship between serum ZAG and MetS in Chinese adults, who diagnosed according to the 2005 International Diabetes Federation (IDF) criteria.

**Methods:** A group of 151 MetS patients, 84 patients with central obesity and 70 healthy controls were enrolled. General clinical information, serum samples were obtained from all subjects and serum ZAG levels were determined via the commercial ELISA kits.

**Results:** Serum ZAG levels were the highest in the control group, then gradually decreased with the severity of the metabolic abnormalities increased (8.78 ± 1.66 μg/mL for control vs. 8.37 ± 1.52 μg/mL for central obesity vs. 7.98 ± 0.94 μg/mL for MetS, *P* < 0.05). It was also decreased progressively with an increasing number of the MetS components (*P* for trend = 0.002). Additionally, serum ZAG/fat mass ratio was calculated and the similar changes were observed in the three groups (0.85 ± 0.53 μg/mL/kg for control vs. 0.39 ± 0.10 μg/mL/kg for central obesity vs. 0.36 ± 0.08 μg/mL/kg for MetS, *P* < 0.05). In the multiple regression analysis, group was a strong independent factor contributing to serum ZAG levels (*P* < 0.001). Furthermore, compared with subjects with the highest tertile of ZAG, subjects in the lowest tertile of ZAG had 1.946-fold higher risk of MetS (95% CI 1.419–6.117, *P* = 0.004). This phenomenon still existed after controlling for age, gender (Model 1), ALP, AST, Cr, UA, Urea based on Model 1 (Model 2), grip strength, smoking, drinking, birth place, current address, education level, manual labor, and exercise frequency based on Model 2 (Model 3). Receiver operation characteristic (ROC) curve analysis revealed that serum ZAG might serve as a candidate biomarker for MetS (sensitivity 57.6%, specificity 70.0% and area under the curve 0.655), and serum ZAG/fat mass ratio showed improved diagnosis value accuracy, with ROC curve area of 0.951 (95% CI, 0.920–0.983, *P* < 0.001), and 90.7% sensitivity and 88.6% specificity.

**Conclusions:** Serum ZAG levels were lowered in patients with MetS and central obesity. The decreased serum ZAG levels were associated with the increased risks of MetS. Serum ZAG, especially serum ZAG/fat mass ratio might be the candidate diagnostic biomarkers for MetS.

## Introduction

Metabolic syndrome (MetS), also known as syndrome X (1), the deadly quartet (2) and the insulin resistance syndrome (3), was firstly described by Kylin in the 1920s (4). MetS is a clustering of risk factors, including central obesity, dyslipidemia, hypertension, and hyperglycemia, that together promote the development of several disorders, including cardiovascular disease (5, 6), type 2 diabetes mellitus (T2DM) (7), cancer (8), non-alcoholic fatty liver disease (NAFLD) (9), chronic kidney disease (10), hyperuricemia and gout (11). Obviously, MetS severely endangers the public health and puts substantial economic burden on the whole society. Unfortunately, due to the global epidemic of obesity and T2DM, the number of people with MetS increases sharply in recent years. Thus, there is an urgent need to take relevant actions and measures to against this serious public health problem.

However, there is even no uniform international definition for the MetS. For now, definitions set out by the World Health Organization (WHO), the National Cholesterol Education Program–Adult Treatment Panel III (NCEP: ATPIII) and the International Diabetes Federation (IDF) were the most widely accepted and clinically used (12). Unlike the other two definitions, central obesity is an obligatory component, but not only one of the key components in the IDF criteria (13). So, it is obvious that obesity, especially central obesity, plays a crucial role in the occurrence and development of the MetS. Central obesity reflects the ectopic fat deposition in visceral adipose tissue, and thus refers to a dysmetabolic state and is predictive of the presence of insulin resistance and other metabolic abnormalities commonly referred to as the MetS (14). Although the pathological mechanisms underlying the close link of obesity and MetS were complex and currently remained largely unknown, the dysfunction of adipose tissue no doubt serves as a major hub (15). Adipose tissue is now recognized as a highly active endocrine organ that could secret multiple biological activity factors called adipokines, which participate in the regulation of systemic metabolism (16). For instance, in studies performed by Li et al. in the Beijing Child and Adolescent Metabolic Syndrome (BCAMS), either the increase of the leptin/adiponectin ratio or serum retinol-binding protein 4 (RBP4) levels has been showed to be associated with the increased risks of the MetS (17, 18).

Zinc-α2-glycoprotein (ZAG) is a 43-kDa soluble glycoprotein firstly isolated from human plasma by Burgi et al. (19). Recent studies identified ZAG as a novel adipokine due to its highly expression in the subcutaneous and visceral white adipose tissue of mice (20) and humans (21) as well as human adipocytes (22). ZAG was initially identified as a lipid-mobilizing factor responsible for the loss of adipose tissue in cancer cachexia (23), and later found to play a vital role in the control of body weight (24, 25). Genetic studies point to ZAG as a candidate gene for body weight regulation since ZAG-deficient mice were susceptible to weight gain (26), whereas transgenic mice overexpressing ZAG exhibited weight loss (27). Animal studies have shown that ZAG gene expression in white adipose tissue was increased in cachectic mice with a profound loss of body weight (28) but decreased in high-fat diet (HFD)-induced obese mice (27, 29) and *ob/ob* mice (30). The negative relationship between ZAG and obesity identified in *in vivo* studies was further verified in human studies. Our previous studies showed that serum ZAG levels were significantly lower in overweight/obese patients and were negatively correlated with body weight, body mass index (BMI) and fat mass after adjustment for age and sex (27, 29). Ge et al. found that ZAG expression in abdominal adipose tissue were negatively associated with visceral fat and sagittal diameter, which further indicates the important role of ZAG expression in central obesity (31). However, Morse et al. studied in obese patients who underwent Roux-En-Y Gastric Bypass (RYGB) surgery found that serum ZAG levels were significantly decreased in obese patients who lost a large amount of weight (32). They speculated that there may be a threshold of BMI at which ZAG is down-regulated, which suggested the protective effect of ZAG during marked weight loss (32).

Up to now, the mechanisms underlying the close link of ZAG and obesity involve the regulation of lipogenesis- and lipolysis-related enzymes, the browning of white adipose tissue and the paracrine manner to stimulate adiponectin production (24, 25). Furthermore, ZAG also play an important role in modulating adipose tissue insulin sensitivity (33, 34). Silencing ZAG resulted in reduced insulin receptor substrate-1 (IRS-1) and glucose transporters-4 (GLUT4) gene expression in primary human adipocytes (34). Indeed, ZAG has emerged as a multifunctional adipokine that involved in the development of various obesity-related disorders, including insulin resistance (33, 35), T2DM (36, 37), hypertension (38, 39), NAFLD (40), polycystic ovary syndrome (PCOS) (41), and Cushing syndrome (42). Given the close link between ZAG and obesity as well as metabolic disorders stated above, it is reasonable to wonder whether ZAG has any relation with the MetS.

So far, only four studies have explored the association between serum ZAG levels and the MetS and the results remained controversial (43–46). Additionally, all of the four studies adopt the MetS criteria approved by the NCEP: ATPIII. Given central obesity is an essential component but not only one of the key components of the MetS in the IDF criteria, and ZAG was closely associated with obesity. It is significant and necessary to investigate serum ZAG levels in the MetS patients, especially in MetS patients diagnosed by using the IDF criteria.

Therefore, the aims of our present study were: (1) to explore serum ZAG levels in 151 MetS patients, 84 patients with central obesity and 70 healthy controls. (2) To explore the associations between serum ZAG levels and the MetS components. (3) To investigate the diagnose power of serum ZAG for discriminating MetS patients from controls.

## Materials and Methods

### Participants

A total of 305 individuals, 151 subjects with MetS, 84 subjects with central obesity and 70 healthy controls were randomly selected from a cross-sectional survey of Han Chinese adults in Guizhou Province in 2012—the National Physical and Health Survey Project of the 12th Five-Year Plan of Science and Technology Support. Based on the 2005 standard criteria of the IDF (47), MetS was diagnosed as the presence of central obesity [waist circumstance (WC) ≥ 90 cm in males and ≥80 cm in females] plus at least two of the following metabolic abnormalities: raised triglycerides (TG) ≥1.7 mmol/L (150 mg/dL) or already have taken lipid-lowering drugs; reduced serum high-density lipoprotein cholesterol (HDL-C) levels, HDL-C <1.03 mmol/L (40 mg/dL) in males and <1.29 mmol/L (50 mg/dL) in females or already have taken drugs; raised blood pressure, systolic blood pressure (SBP) ≥130 mmHg or diastolic blood pressure (DBP) ≥85 mmHg, or already have taken antihypertensive drugs; raised fasting blood glucose (FBG) levels, FBG ≥5.6 mmol/L (100 mg/dL), or previously diagnosed T2DM. Subjects in the central obesity group were also central-obese, but only with 0–1 abnormal metabolic component stated above. Age-gender matched subjects with normal WC and metabolic components as well as normal liver and kidney functions that assessed by the routine blood tests were used as the healthy controls. Our study was approved by the Ethics Committee of Basic Medical Sciences Institute of Chinese Academy of Medical Sciences (No. 028-2013), and written informed consent documents were collected from all individuals before they entering the study.

### Clinical and Anthropometric Measurements

All participants underwent anthropometric measurements and filled in the medical questionnaires. In our current study, anthropometric measurements were performed when all subjects were wearing light clothing and no shoes. Body weight, fat mass, lean muscle mass, and body fat rate were measured by using a bioelectric-impedance analyzer (BC-420, Tanita, Tokyo, Japan). WC was measured at the midpoint between the iliac crest and the lowest margin of the ribs by using a cloth measuring tape (48). Hip circumference (HC) was taken by wrapping a cloth measuring tape around the maximum circumference of the hips. BMI, waist-to-hip ratio (WHR) and fat-to-muscle ratio were calculated as weight (kg)/height (m^2^), WC (cm)/HC (cm), and fat mass (kg)/lean muscle mass (kg), respectively. Hand grip strength was measured three times and the mean value was recorded. Blood pressure was measured three times with participants seated after at least 5 min of rest by using an electronic sphygmomanometer (HEM-907, Omron Healthcare, Kyoto, Japan). The values of the three measurements were taken with at least 1 min interval and the mean values were recorded. All participants were also asked to fill the questionnaires that included questions on disease history, current smoking or drinking status, birth or current address, education level, manual labor, and exercise frequency. Disease history included the diagnosis and treatment of hypertension and diabetes mellitus. Education level was expressed as Junior high school or below and High school or above. Manual labor was expressed as mild, moderate and heavy. Exercise frequency was expressed as 0, <3 times/month, 1–2, 3–4, and 5–7 times/week.

### Blood Sampling and Biochemical Measurements

After overweight fasting (about 10–12 h), Venous blood samples were collected from the antecubital vein of all subjects. After centrifugation (3,000 rpm, 10 min), serum samples were obtained and stored at −80°C in aliquots. Total protein (TP), albumin (ALB), total cholesterol (TC), TG, HDL-C, low-density lipoprotein cholesterol (LDL-C), gamma-glutamyl transpeptidase (GGT), aspartate transaminase (AST), FBG, creatinine (Cr), uric acid (UA), alkaline phosphatase (ALP), and urea nitrogen (Urea) were measured through an automatic biochemical analyzer according to standard procedures in our clinical laboratory (Beckman Company AU5800, USA). Fasting insulin (FINS) concentration was measured via the Siemens Centaur XP system (Siemens, Tarrytown, USA). Homeostasis model assessment estimate of insulin resistance (HOMA-IR) was calculated using the following equation: HOMA-IR = FINS (mU/L) × FBG (mmol/L)/22.5 (49). Serum ZAG levels were determined using commercial enzyme linked immunosorbent assay (ELISA) kits following the manufacturer's protocol (SEL231Hu, USCN Life Science Inc. Wuhan, China). Serum samples were diluted 100-fold before the assay. The detectable range of the kit was 4.7–300 ng/mL and the minimum detectable dose of human ZAG was 1.8 ng/mL. The intra- and inter-assay variations were 7.40 and 11.97%, respectively.

### Statistical Analysis

Results were shown as mean ± standard deviation (SD) or percentage, as appropriate. All skewed distributions were natural logarithm (ln) transformed for analysis. The normality of continuous data was tested via Shapiro-Wilk test and *P* > 0.05 was considered as normal distribution. The distribution of serum ZAG levels was skewed and therefore naturally logarithmic transformed before analysis. One-way ANOVA and Dunnett's T3 *post-hoc* test were used for the comparison of continuous parameters in different groups. Chi-square or Kruskal-Wallis test was used for the comparison of categorical variables, such as gender, birth place, current address, education level, manual labor, and exercise frequency. Bivariate correlations between serum ZAG levels and other parameters were analyzed by Pearson's correlation analysis. Multiple regression analysis was performed with naturally logarithmic transformed serum ZAG as dependent variable and group, age, gender, BMI, WHR, fat-to-muscle ratio, body fate rate, grip strength, DBP, ALP, Cr, and exercise frequency as independent variables. Unconditional logistic regression analysis was conducted to explore the odds ratio (OR) and 95% confidence intervals (CIs) of serum ZAG for MetS/central obesity risks. Subjects in the MetS and control groups as well as those in the central obesity and control groups were divided into tertiles according to the serum ZAG levels. ORs and 95% CIs for the lower two categories were calculated with the highest one as a reference. For models investigating the relationship between serum ZAG levels and MetS/central obesity risks, we adjusted for age and gender (male and female) in Model 1, and further adjusted for ALP, ALT, Cr, UA, and Urea in Model 2, with further adjustment for grip strength, smoking, drinking, birth place, current address, education level, manual labor, and exercise frequency in Model 3. The area under the receiver operating characteristic (ROC) curve (AUC) was used to assess the diagnostic value of serum ZAG or serum ZAG/fat mass ratio for the MetS. All statistical analyses were conducted using SPSS version 20.0 software for Windows (SPSS Inc., Chicago, IL, USA). All data graphing was performed by using GraphPad Prism 7.0 (GraphPad software Inc., La Jolla, CA, USA). *P* < 0.05 was considered as statistical significance.

## Results

### General Characteristics of the Study Participants

General characteristics, including anthropometric parameters, metabolic parameters and personal information of all subjects in the three groups were shown in Table 1. As expected, fat mass, SBP, DBP, TG, GGT, FBG, and HOMA-IR were gradually increased while HDL-C was gradually decreased among the control, the central obesity, and the MetS groups (*P* all < 0.05). Subjects in the MetS group had higher body weight, BMI, WC, HC, WHR, lean muscle mass, fat-to-muscle ratio, body fat fate, ALB, TC, LDL-C, AST, FINS, UA, hypertension (%), diabetes (%), and current address in cities (%) than the control group, and higher TC, UA and ALP levels than the central obesity group (*P* all < 0.05). Body weight, BMI, WC, HC, WHR, lean muscle mass, fat-to-muscle ratio, body fat rate, LDL-C, FINS, and mild manual labor (%) were significantly higher in the central obesity group than in the control group (*P* all < 0.05). In addition, males in the MetS and the central obesity groups had significantly higher WC and HC than females (*P* all < 0.05). Males in each group had significantly higher WHR than females (*P* < 0.05). No significant differences were found in age, gender, body height, grip strength, TP, Cr, Urea, smoking, drinking, birth place, education level, and exercise frequency (*P* all > 0.05).

**Table 1:** General characteristics of all subjects according to study groups.

**Variables**	**Control group (*n* = 70)**	**Central obesity group (*n* = 84)**	**MetS group (*n* = 151)**	***P*-value**
**Anthropometric parameters**				
Age (year)	47.81 ± 8.56	47.56 ± 10.36	49.51 ± 9.04	0.227
Gender				0.109
Male	37/70 (52.86%)	33/84 (39.29%)	58/151 (38.41%)	
Female	33/70 (47.14%)	51/84 (60.71%)	93/151 (61.59%)	
Body weight (kg)	53.42 ± 8.64	66.63 ± 8.68^a^	68.43 ± 10.35^a^	**<0.001**
Body height (m)	157.75 ± 8.13	157.89 ± 8.73	157.90 ± 9.41	0.992
BMI (kg/m^2^)	21.40 ± 2.65	26.66 ± 2.28^a^	27.33 ± 2.64^a^	**<0.001**
WC (cm)				
Male	73.82 ± 8.46	94.18 ± 3.30^a^	95.55 ± 4.17^a^	**<0.001**
Female	70.92 ± 6.68	86.19 ± 5.54^a^^c^	88.61 ± 7.15^a^^b^^c^	**<0.001**
HC (cm)				
Male	83.65 ± 5.24	94.62 ± 4.07^a^	94.75 ± 3.29^a^	**<0.001**
Female	83.85 ± 5.67	91.56 ± 4.30^a^^c^	91.78 ± 5.81^a^^c^	**<0.001**
WHR				
Male	0.88 ± 0.06	1.00 ± 0.04^a^	1.01 ± 0.04^a^	**<0.001**
Female	0.85 ± 0.06^c^	0.94 ± 0.06^a^^c^	0.97 ± 0.05^a^^b^^c^	**<0.001**
Fat mass (kg)	12.60 ± 4.87	21.95 ± 3.63^a^	23.33 ± 4.65^a^^b^	**<0.001**
Lean muscle mass (kg)	38.65 ± 7.00	42.24 ± 8.29^a^	42.63 ± 8.96^a^	**0.004**
Grip strength (kg)	30.55 ± 9.33	31.11 ± 8.91	30.88 ± 9.40	0.933
Fat-to-muscle ratio	0.34 ± 0.15	0.54 ± 0.15^a^	0.57 ± 0.16^a^	**<0.001**
Body fat rate (%)	23.41 ± 8.00	33.35 ± 5.98^a^	34.43 ± 6.17^a^	**<0.001**
SBP (mmHg)	115.01 ± 8.75	126.95 ± 16.57^a^	140.68 ± 18.23^a^^b^	**<0.001**
DBP (mmHg)	69.76 ± 7.87	77.51 ± 9.83^a^	86.23 ± 10.91^a^^b^	**<0.001**
**Metabolic parameters**				
TP (g/L)	69.33 ± 5.65	70.50 ± 6.37	70.77 ± 6.73	0.289
ALB (g/L)	39.19 ± 3.99	40.38 ± 3.88	40.68 ± 4.29^a^	**0.041**
TC (mmol/L)	4.77 ± 0.87	5.01 ± 0.88	5.31 ± 1.18^a^^b^	**0.001**
TG (mmol/L)	0.96 ± 0.31	1.40 ± 0.77^a^	3.08 ± 1.98^a^^b^	**<0.001**
HDL-C (mmol/L)	1.62 ± 0.28	1.46 ± 0.27^a^	1.23 ± 0.24^a^^b^	**<0.001**
LDL-C (mmol/L)	2.69 ± 0.73	3.02 ± 0.73^a^	3.07 ± 0.79^a^	**0.002**
GGT (U/L)	18.63 ± 15.34	35.33 ± 46.06^a^	55.29 ± 78.91^a^^b^	**<0.001**
AST (U/L)	21.77 ± 6.97	24.36 ± 7.56	27.33 ± 12.62^a^	**0.001**
FBG (mmol/L)	4.75 ± 0.40	4.94 ± 0.56^a^	5.57 ± 1.16^a^^b^	**<0.001**
FINS (mU/L)^*^	5.41 ± 2.67	9.99 ± 5.95^a^	11.71 ± 6.63^a^	**<0.001**
HOMA-IR	1.15 ± 0.58	2.23 ± 1.53^a^	3.03 ± 2.36^a^^b^	**<0.001**
Cr (μmol/L)	71.96 ± 13.92	71.63 ± 15.06	72.02 ± 15.73	0.982
UA (μmol/L)	279.09 ± 84.74	304.39 ± 89.15	342.62 ± 88.86^a^^b^	**<0.001**
ALP (U/L)	69.83 ± 21.56	68.25 ± 22.12	75.89 ± 23.20^b^	**0.026**
Urea (mmol/L)	5.06 ± 1.43	4.68 ± 1.22	4.85 ± 1.32	0.203
**Personal information**				
Hypertension (%)	0	7/83 (8.43%)	34/151 (22.52%)^a^	**<0.001**
Diabetes (%)	0	1/83 (1.20%)	9/151 (5.96%)^a^	**0.031**
Smoking (%)	28/69 (40.58%)	22/83 (26.51%)	38/151 (25.17%)	0.055
Drinking (%)	21/70 (30.00%)	31/83 (37.35%)	56/151 (37.09%)	0.545
Birth place				0.300
Cities	18/70 (25.71%)	29/83 (34.94%)	54/150 (36.00%)	
Villages	52/70 (74.29%)	54/83 (65.06%)	96/150 (64.00%)	
Current address				**0.029**
Cities	33/70 (47.14%)	49/83 (59.04%)	99/150 (66.00%)^a^	
Villages	37/70 (52.86%)	34/83 (40.96%)	51/150 (34.00%)	
Education level				0.081
Junior high school or below	47/69 (68.12%)	46/83 (55.42%)	105/151 (69.54%)	
High school or above	22/69 (31.88%)	37/83 (44.58%)	46/151 (30.46%)	
Manual labor				**0.006**
Mild	36/70 (51.43%)	64/83 (77.11%)^a^	101/151 (66.89%)	
Moderate	15/70 (21.43%)	8/83 (9.64%)^a^	18/151 (11.92%)	
Heavy	19/70 (27.14%)	11/83 (13.25%)^a^	32/151 (21.92%)	
Exercise frequency (day/week)				0.301
0	48/69 (69.57%)	49/83 (59.04%)	91/149 (61.07%)	
Monthly <3	2/69 (2.90%)	2/83 (2.41%)	10/149 (6.71%)	
1–2	4/69 (5.80%)	5/83 (6.02%)	8/149 (5.37%)	
3–4	7/69 (10.14%)	10/83 (12.05%)	13/149 (8.72%)	
5–7	8/69 (11.59%)	17/83 (20.48%)	27/149 (18.12%)	
**Adipokine levels**				
ZAG (μg/mL)^*^	8.78 ± 1.66	8.37 ± 1.52^a^	7.98 ± 0.94^a^^b^	**<0.001**
ZAG/fat mass (μg/mL/kg)	0.85 ± 0.53	0.39 ± 0.10^a^	0.36 ± 0.08^a^^b^	**<0.001**

*Continuous variables were expressed as mean ± SD. Categorial variables were expressed as n (%). BMI, body mass index; WC, waist circumference; HC, hip circumference; WHR, waist-to-hip ratio; SBP, systolic blood pressure; DBP, diastolic blood pressure; TP, total protein; ALB, albumin; TC, total cholesterol; TG, triglycerides; HDL-C, high-density lipoprotein cholesterol; LDL-C, low-density lipoprotein cholesterol; GGT, gamma-glutamyl transpeptidase; AST, aspartate transaminase; FBG, fasting blood glucose; FINS, fasting insulin; HOMA-IR, homeostasis model assessment estimate of insulin resistance; Cr, creatinine; UA, uric acid; ALP, alkaline phosphatase; Urea, urea nitrogen; ZAG, zinc-a2-glycoprotein; MetS, metabolic syndrome. The P-value column was the result of comparison among the three groups*.

a *P < 0.05 compared with the control group*;

b *P < 0.05 compared with the central obesity group*;

c *P < 0.05 compared with males in corresponding group. Bold font indicated P < 0.05*.

* *Variables were ln-transformed before analysis*.

### Serum ZAG Levels and ZAG/Fat Mass Ratio in the MetS, Central Obesity and Control Groups

**Figure 1:**
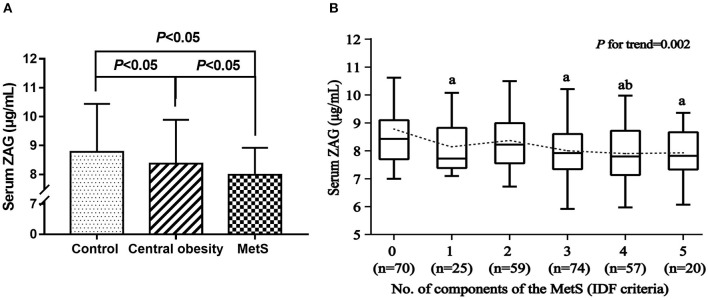
**(A)** Serum ZAG levels in MetS, central obesity patients and controls. **(B)** Serum ZAG levels in relation to the number of MetS components. All values are expressed as the mean ± SD. MetS: metabolic syndrome; ZAG: zinc-α2-glycoprotein. ^a^*P* < 0.05 compared with the MetS component number = 0; ^b^*P* < 0.05 compared with the MetS component number = 2.

As displayed in Table 1 and Figure 1A, serum ZAG levels were significantly lower in the MetS group than in the central obesity group (7.98 ± 0.94 μg/mL vs. 8.37 ± 1.52 μg/mL, *P* < 0.05) and the control group (7.98 ± 0.94 μg/mL vs. 8.78 ± 1.66 μg/mL, *P* < 0.05). Serum ZAG levels in the central obesity group were significantly lower in comparison with the control group (8.37 ± 1.52 μg/mL vs. 8.78 ± 1.66 μg/mL, *P* < 0.05). To further explore the relationship between serum ZAG levels and the MetS, we stratified serum ZAG by the number of MetS components. As depicted in Figure 1B, serum ZAG levels decreased progressively with increasing number of MetS components (*P* for trend = 0.002). Additionally, serum ZAG levels in the three groups were also explored according to gender. However, no significant difference was found in serum ZAG levels in each group with respect to gender (Supplementary Figure 1).

Because ZAG was produced abundantly by white adipose tissue (24), serum ZAG/fat mass ratio was calculated and analyzed in the three groups. As presented in Table 1, serum ZAG/fat mass ratio were the highest in the control group, then gradually decreased with the severity of metabolic abnormalities increasing. When compared with the control group, ZAG/fat mass ratio in the central obesity and the MetS groups were notably decreased by 54.12 and 57.65%, respectively (0.39 ± 0.10 μg/mL/kg and 0.36 ± 0.08 μg/mL/kg vs. 0.85 ± 0.53 μg/mL/kg, *P* all < 0.05). Additionally, ZAG/fat mass ratio in the MetS group were further decreased in comparison with that in the central obesity group (0.39 ± 0.10 μg/mL/kg vs. 0.36 ± 0.08 μg/mL/kg, *P* < 0.05).

### Correlations and Regression of Serum ZAG Levels With Clinical Parameters in the Study Population

As shown in Table 2, bivariate correlation analysis was performed to investigate the relationships between serum ZAG levels and various clinical parameters. Serum ZAG levels were positively associated with HDL-C (r = 0.186), but negatively associated with body weight (r = −0.118), BMI (r = −0.170), WC (r = −0.168), HC (r = −0.114), WHR (r = −0.168), fat mass (r = −0.168), fat-to-muscle ratio (r = −0.129), body fat rate (r = −0.128), and TG (r = −0.140) in all subjects (*P* all < 0.05). In the control group, serum ZAG levels were only found to be negatively associated with exercise frequency (r = −0.290, *P* < 0.05). In the MetS subjects, serum ZAG levels were positively associated with TC (r = 0.170), HDL-C (r = 0.162), Cr (r = 0.212), and Urea (r = 0.161) (*P* all < 0.05). There were no significant relationships were found between serum ZAG levels and other clinical variables (*P* > 0.05).

**Table 2:** Bivariate correlation between serum ZAG levels and other parameters.

	Serum ZAG levels (μg/mL)^*^			
**Variables**	**All subjects**	**Control group**	**Central obesity group**	**MetS group**
Age (year)	0.022	−0.030	0.021	0.109
Male/female	−0.040	0.113	−0.042	−0.076
Body weight (kg)	**−0.118**	−0.013	0.086	−0.022
Body height (m)	0.022	−0.067	0.072	0.046
BMI (kg/m^2^)	**−0.170**	0.054	0.045	−0.096
WC (cm)	**−0.168**	0.016	0.117	−0.073
HC (cm)	**−0.114**	0.085	0.046	−0.024
WHR	**−0.168**	−0.046	0.097	−0.085
Fat mass (g)	**−0.168**	0.112	0.020	−0.101
Lean muscle mass (g)	−0.031	−0.090	0.076	0.027
Grip strength (kg)	−0.069	−0.235	−0.052	0.024
Fat-to-muscle ratio	**−0.129**	0.165	−0.036	−0.093
Body fat rate (%)	**−0.128**	0.152	−0.041	−0.092
SBP (mmHg)	−0.077	−0.028	0.089	0.108
DBP (mmHg)	−0.046	0.018	0.181	0.135
TP (g/L)	0.018	0.124	−0.061	0.064
ALB (g/L)	0.003	0.088	−0.084	0.088
TC (mmol/L)	0.034	−0.022	0.058	**0.170**
TG (mmol/L)	**−0.140**	0.127	−0.040	−0.019
HDL-C (mmol/L)	**0.186**	−0.063	0.069	**0.162**
LDL-C (mmol/L)	0.029	−0.015	0.101	0.125
GGT (U/L)	0.001	−0.096	0.162	0.063
AST (U/L)	−0.009	−0.102	0.189	0.052
FBG (mmol/L)	−0.099	−0.172	0.124	−0.018
FINS (mU/L)	−0.078	−0.023	0.038	0.029
HOMA-IR	−0.078	−0.049	0.051	0.017
Cr (μmol/L)	0.077	−0.098	0.039	**0.212**
UA (μmol/L)	−0.027	−0.043	0.015	0.127
ALP (U/L)	0.091	0.138	0.129	0.127
Urea (mmol/L)	0.049	0.028	−0.147	**0.161**
Birth place	−0.031	−0.164	−0.008	−0.022
Current address	−0.066	−0.159	−0.081	−0.096
Education level	0.090	0.199	0.063	0.051
Manual labor	0.049	−0.037	0.014	0.084
Exercise frequency (day/week)	−0.087	**−0.290**	0.030	−0.091

*Pearson's correlation tests were used. BMI, body mass index; WC, waist circumference; HC, hip circumference; WHR, waist-to-hip ratio; SBP, systolic blood pressure; DBP, diastolic blood pressure; TP, total protein; ALB, albumin; TC, total cholesterol; TG, triglycerides; HDL-C, high-density lipoprotein cholesterol; LDL-C, low-density lipoprotein cholesterol; GGT, gamma-glutamyl transpeptidase; AST, aspartate transaminase; FBG, fasting blood glucose; FINS, fasting insulin; HOMA-IR, homeostasis model assessment estimate of insulin resistance; Cr, creatinine; UA, uric acid; ALP, alkaline phosphatase; Urea, urea nitrogen; ZAG, zinc-a2-glycoprotein. Bold font indicated P < 0.05*.

* *Variables were ln-transformed before analysis*.

**Figure 2:**
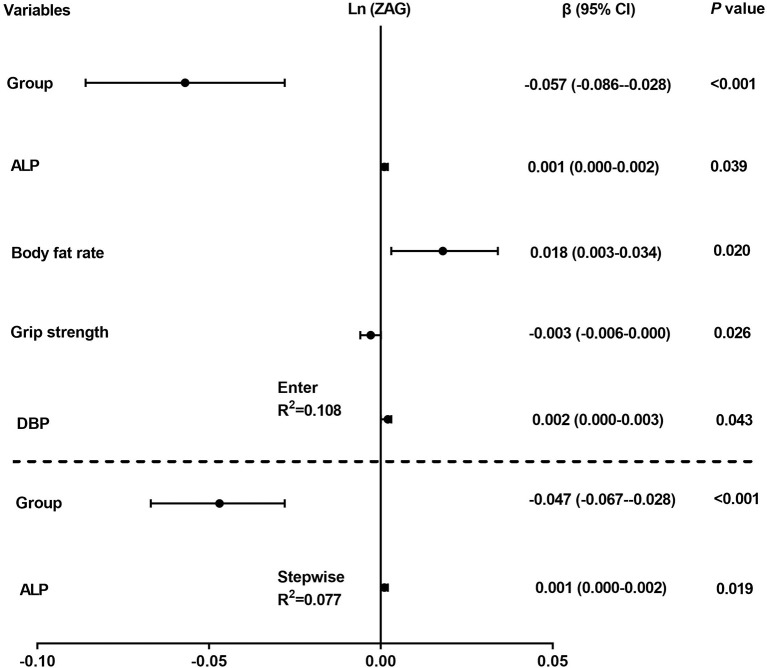
Multiple regression analysis of variables independently related to serum ZAG levels in all subjects. The regression coefficients (β) and 95% confident interval (CI) from linear regression analysis were displayed.

Then multiple regression analysis was used to explore independent variables contributing to serum ZAG levels. As demonstrated in Figure 2, all factors enter and stepwise regression analyses showed that group was an independent factor affecting serum ZAG levels, which was consistent with the results in Figure 1. Additionally, ALP also was an independent variable contributing to serum ZAG levels.

### Association of Serum ZAG Levels With MetS/Central Obesity Risks

In order to further explain the association between serum ZAG levels and MetS/central obesity risks, subjects in the MetS and control groups were stratified into three parts according to serum ZAG tertiles (lowest: <7.60 μg/mL; median: 7.60–8.60 μg/mL; highest: ≥8.60 μg/mL). As displayed in Table 3, univariate unconditional logistic regression analysis showed that subjects in the lowest tertile of ZAG levels had 1.946-fold higher risk of MetS when compared with those in the highest tertile levels (OR = 2.946, 95% CI 1.419–6.117, *P* = 0.004). After adjusting the age and gender in Model 1, the tendency still existed with an OR of 2.963 (95% CI 1.413–6.211, *P* = 0.004) in the lowest tertile of ZAG levels. When further controlling ALP, AST, Cr, UA, and Urea in Model 2, the increased OR of MetS in the lowest ZAG levels persisted and higher than in Model 1 (OR = 4.217, 95% CI 1.716–10.365, *P* = 0.002). In Model 3, even after adjusting for grip strength, smoking, drinking, birth place, current address, education level, manual labor, and exercise frequency, the significantly increased risk of MetS was also observed in subjects with the lowest ZAG levels and higher than in Model 2 (OR = 6.124, 95% CI 1.992–18.830, *P* = 0.002). Similarly, subjects in central obesity/controls were also divided into three parts based on serum ZAG tertiles (lowest: <7.72 μg/mL; median: 7.72–8.72 μg/mL; highest: ≥8.72 μg/mL). However, no significant associations were found between serum ZAG levels and central obesity risk as shown in Table 3.

**Table 3:** Unconditional logistic regression analysis of MetS/central obesity risks according to the tertiles of serum ZAG levels.

	**ZAG tertiles**		
**Measurement**	**Lowest OR (95% CI)**	**Median OR (95% CI)**	**Highest OR (95% CI)**
**ZAG in MetS vs. control group**			
Range (μg/mL)	<7.60	≥7.60–8.60	≥8.60
MetS/controls	58/15	51/23	42/32
Univariate (*R*^2^ = 0.055)	**2.946 (1.419–6.117)**	1.689 (0.861-3.314)	1 (reference)
*P-*value	**0.004**	0.127	
Model 1 (*R*^2^ = 0.089)	**2.963 (1.413–6.211)**	1.744 (0.879–3.461)	1 (reference)
*P-*value	**0.004**	0.112	
Model 2 (*R*^2^ = 0.392)	**4.217 (1.716–10.365)**	1.815 (0.804–4.100)	1 (reference)
*P-*value	**0.002**	0.151	
Model 3 (*R*^2^ = 0.552)	**6.124 (1.992–18.830)**	**3.126 (1.076–9.086)**	1 (reference)
*P-*value	**0.002**	**0.036**	
**ZAG in central obesity vs. control group**			
Range (μg/mL)	<7.72	≥7.72 to 8.72	≥8.72
Central obesity/controls	33/18	25/27	26/25
Univariate (*R*^2^ = 0.028)	1.763 (0.796–3.902)	0.890 (0.411–1.928)	1 (reference)
*P-*value	0.162	0.768	
Model 1 (*R*^2^ = 0.052)	1.826 (0.817–4.079)	0.930 (0.425–2.035)	1 (reference)
*P-*value	0.142	0.855	
Model 2 (*R*^2^ = 0.187)	1.911 (0.806–4.533)	0.935 (0.402–2.171)	1 (reference)
*P-*value	0.142	0.875	
Model 3 (*R*^2^ = 0.333)	2.178 (0.794–5.974)	1.099 (0.406–2.978)	1 (reference)
*P-*value	0.130	0.852	

*ZAG, zinc-a2-glycoprotein; MetS, metabolic syndrome. Multivariate odds ratios (OR) and 95% confidence intervals (CI) from unconditional logistic regression models were used in the analysis. Model 1: adjusted for age and gender. Model 2: adjusted for Model 1+ alkaline phosphatase, aspartate transaminase, creatinine, uric acid, urea nitrogen. Model 3: adjusted for Model 2+ grip strength, smoking, drinking, birth place, current address, education level, manual labor, and exercise frequency. Bold font indicated P < 0.05*.

### Diagnostic Value of Serum ZAG Levels and ZAG/Fat Mass Ratio for MetS Risk

**Figure 3:**
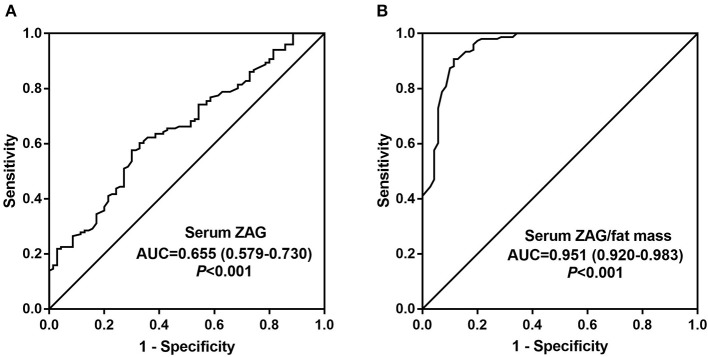
Comparison for ROC curve analysis of serum ZAG **(A)** and serum ZAG/fat mass ratio **(B)** in MetS patients and controls. ROC curves were derived by plotting the relationship between the specificity and the sensitivity at various cut off levels. ZAG, zinc-α2-glycoprotein; ROC, receiver-operating characteristic; AUC, area under the curve.

Finally, to explore the diagnostic value of ZAG for MetS, ROC curve analysis was performed. As illustrated in Figure 3A, serum ZAG might serve as a candidate biomarker for distinguishing the MetS patients from controls with the AUC was 0.655 (95% CI 0.579–0.730, *P* < 0.001), a sensitivity of 57.6% and a specificity of 70.0%. Moreover, ZAG/fat mass ratio showed improved diagnosis value accuracy, with ROC curve area of 0.951 (95% CI, 0.920–0.983, *P* < 0.001), and 90.7% sensitivity and 88.6% specificity (Figure 3B).

## Discussion

ZAG was a multifunctional adipokine that played a crucial role in the development of obesity and its associated disorders. Our previous studies have demonstrated the significantly decreased serum ZAG levels in overweight/obese patients and its negative association with body weight, BMI and fat mass (27, 29). Moreover, serum ZAG levels have been found to be decreased in various obesity-related disorders, including T2DM (36), hypertension (39), premature coronary artery disease (50), and PCOS (41), which further indicates the pivotal role of ZAG in the regulation of metabolism. In our present study, we found that serum ZAG levels were significantly lower in MetS patients in comparison with those in the central obesity group and control group, and serum ZAG in central obesity group were also lower than that in the control group. In line with our results, previous studies performed by Lei et al. also found the significantly lower serum ZAG levels in MetS patients (46). However, to our surprise, Stejskal et al. studied in a total of 228 Czechs found that there was no significant difference in serum ZAG levels between MetS patients and healthy controls (43). The difference between the races may partly explain the discrepancy of serum ZAG levels in MetS patients. Additionally, Stejskal et al. studied in elderly MetS patients (63.0 ± 11.5 y), while the MetS patients recruited in our present studies were relatively younger (49.51 ± 9.04 y). Previous studies have demonstrated that serum ZAG levels were correlated positively with age (44). Thus, the different age of the two studies may also partly account for the discrepant findings in the association of ZAG with the MetS. In present study, we were the first to explore serum ZAG in MetS patients diagnosed by the IDF criteria, which included central obesity as one of the essential components. Given the close link between ZAG and obesity observed in animal experiments and clinical studies (20, 21, 26, 27, 29, 51), the MetS diagnostic criteria adopt in our present studies may better reveal the potential role of ZAG in pathogenesis of the MetS.

Our present studies also found that serum ZAG levels decreased progressively with the increasing number of the MetS components. However, inconsistent with our results, Yeung et al. studied in Hong Kong populations (44) and Tsai et al. studied in Taiwan populations (45) reported that serum ZAG levels elevated progressively with an increasing number of the MetS components. The discrepancy may be due to the following reasons. Yeung et al. studied in subjects with BMI at 25.4 ± 4.1 kg/m^2^, which was a little smaller than ours (27.33 ± 2.64 kg/m^2^) and covered a wider range of adiposity that randomly selected from the population-based Hong Kong Cardiovascular Risk Factor Prevalence Study, the “paradoxical” elevation of serum ZAG might be a compensatory upregulation of the human body to counteract the metabolic stress imposed by early mild obesity (44). While the studies performed by Tsai et al. mainly focused on the effects of smoking on ZAG levels and its relationship with the MetS. As the expression of ZAG in the airway epithelium was enhanced in chronic smokers (52), the elevation of serum ZAG might partly derive from the airway epithelium in addition from the adipose tissue (45). ZAG is a multifunctional adipokine that involved in the development of various obesity-related disorders (25, 53). Our previous studies have shown that ZAG overexpression could reduce the body weight of HFD-induced obese mice by regulation of lipogenesis- and lipolysis-related enzymes (27) and promoting the browning of white adipose tissue (29). Furthermore, ZAG also play an important role in modulating adipose tissue insulin sensitivity (33, 34). Silencing ZAG in primary human adipocytes resulted in the reduced IRS-1 and GLUT4 gene expression, indicating the role of ZAG in glucose uptake and insulin action (34). All these findings together with our present results suggest the important role of ZAG in the regulation of metabolism.

Additionally, the independent contribution of group (MetS, central obesity, control) to serum ZAG levels observed in our results by multiple linear regression analysis further confirmed the close relationship between serum ZAG and the MetS. Moreover, univariate unconditional logistic regression analysis displayed that subjects in the lowest tertile of ZAG levels had 1.946-fold higher risk of MetS than those in the highest ZAG tertiles. The significantly increased risk of MetS was also observed and even higher in the lowest ZAG levels after adjusting the age and gender in Model 1, and further controlling ALP, AST, Cr, UA, Urea in Model 2, as well as adjusting for grip strength, smoking, drinking, birth place, current address, education level, manual labor, exercise frequency in Model 3. These results raise the possibility that the decreased serum ZAG levels might act as a biomarker of the MetS. Further analyses using ROC curves found that serum ZAG might be a candidate biomarker for the MetS. ZAG could discriminate the MetS patients from controls with the AUC of 0.655, the sensitivity of 57.6% and the specificity of 70.0%. In support of our results, Lei et al. studied in a cohort of 489 Chinese individuals reported that serum ZAG was a useful predictor for the MetS with the AUC of 0.80, the sensitivity of 92.0% and the specificity of 59.0% (46). However, the diagnostic role of ZAG for the MetS still need to be verified in large-scale prospective studies in the future, especially in other ethnic groups and in MetS patients diagnosed by the WHO and NCEP: ATPIII criteria.

It is worth noting that in our present study serum ZAG/fat mass ratio were the highest in the control group, then gradually decreased in the central obesity and the MetS groups, which was in consistent with the variation trend of serum ZAG in the three groups. ZAG is a novel adipokine that can be highly expression in the subcutaneous and visceral white adipose tissue of mice (20, 21). Various studies have confirmed the reduced ZAG gene expression in adipose tissue and the decreased serum ZAG levels from obese persons (27, 51, 54). Moreover, ROC curve analysis showed that serum ZAG/fat mass ratio had improved diagnosis value accuracy than serum ZAG levels, with ROC curve area of 0.951 and 90.7% sensitivity and 88.6% specificity. These findings suggest that serum ZAG/fat mass ratio also could be useful for identifying MetS patients from controls.

In the present studies, no significant difference in serum ZAG levels was found between males and females. Inconsistent with our results, previous studies by Yeung et al. performed in 258 subjects randomly selected from the Hong Kong Cardiovascular Risk Factor Prevalence Study have showed the significantly higher serum ZAG levels in males than that in females (44). In addition, we did not find any significant association between serum ZAG levels and gender in the multiple regression analysis. The results were not agreement with our recently studies in premature coronary artery disease patients (50) and Selva et al. studies in simple obese patients (21), which found that gender was an independent factor affecting serum ZAG levels. In a word, current studies were quite few and the results remained controversial. Whether sex hormones regulate ZAG expression or not warrants further investigation.

Additionally, we found that ALP was an independent factor associated with serum ZAG levels. ALP are dimeric hydrolytic enzymes that 95% of plasma ALP are synthetized in liver and bone by tissue non-specific alkaline phosphatase (AP-TNAP) gene (55). Recently, Hernández-Mosqueira et al. demonstrated that AP-TNAP also expressed in the adipose tissue and was involved in the adipokine synthesis and secretion (55). Our present study is the first to found the relationship between serum ZAG levels and ALP. The association between serum ZAG and ALP still need to be verified in the future studies, especially in the MetS patients.

There are some limitations in our study. Firstly, due to the cross-sectional design of our current study, it is still unknown the causal relationships between serum ZAG levels and the MetS. Longitudinal intervention studies still need to be done in the future. Secondly, the diagnostic value of ZAG for the MetS observed in our studies need to be verified in another prospective study, especially in other ethnic groups and in MetS patients diagnosed by the WHO and NCEP: ATPIII criteria. Thirdly, serum ZAG levels were firstly explored in MetS patients diagnosed by the IDF criteria. However, our present studies only included Chinese people with small sample size. Therefore, the extrapolation of serum ZAG in MetS patients diagnosed by the IDF criteria should be undertaken in other ethnic groups with large-scale. Despite these limitations, our study has a number of strengths. This is the first study to investigate serum ZAG levels in the MetS patients diagnosed by the IDF criteria. Unlike the previous studies, central obesity in our present study is an obligatory component, but not only one of the key components for the MetS. Thus, our study can help to identify the role of central obesity in the relationship between ZAG and the MetS.

## Conclusion

In summary, our present found that serum ZAG levels were significantly lowered in patients with MetS and central obesity, according to the IDF criteria. The decreased serum ZAG levels were associated with the increased risks of MetS, even after controlling other confounding variables. Serum ZAG, especially serum ZAG/fat mass ratio might be the candidate diagnostic biomarkers for MetS.

## Data Availability Statement

The datasets generated for the present study are available on request to the corresponding author.

## Ethics Statement

The studies involving human participants were reviewed and approved by the Ethics Committee of Basic Medical Sciences Institute of Chinese Academy of Medical Sciences (No. 028-2013). The patients/participants provided their written informed consent to participate in this study.

## Author Contributions

LW designed the experiments and revised the primary manuscript. ML did the statistical analysis and wrote the primary manuscript. DN performed the measurement of serum ZAG levels. HZ and GS contributed to recruitment of patients and the collection of the clinical data. DW, BP, YY, HY, and KY collected the serum samples and finished the clinical and biochemical parameters measurements. HP and FG designed the experiment, supervised the whole study, and revised the primary manuscript.

### Conflict of Interest

The authors declare that the research was conducted in the absence of any commercial or financial relationships that could be construed as a potential conflict of interest.

## Abbreviations

ALB: albumin
ALP: alkaline phosphatase
AP-TNAP: tissue non-specific alkaline phosphatase
AST: aspartate transaminase
AUC: area under the curve
BCAMS: Beijing Child and Adolescent Metabolic Syndrome
BMI: body mass index
CIs: confidence intervals
Cr: creatinine
DBP: diastolic blood pressure
DECODE: Diabetes Epidemiology: Collaborative Analysis of Diagnostic Criteria in Europe
ELISA: enzyme linked immunosorbent assay
FBG: fasting blood glucose
FINS: fasting insulin
GGT: gamma-glutamyl transpeptidase
HC: hip circumference
HDL-C: high-density lipoprotein cholesterol
HFD: high-fat diet
HOMA-IR: homeostasis model assessment estimate of insulin resistance
IDF: International Diabetes Federation
IRAS: Insulin Resistance Atherosclerosis Study
LDL-C: low-density lipoprotein cholesterol
MetS: metabolic syndrome
NAFLD: non-alcoholic fatty liver disease
NCEP: ATPIII: National Cholesterol Education Program–Adult Treatment Panel III
Central obesity: non-metabolic syndrome
OR: odds ratio
PCOS: polycystic ovary syndrome
RBP4: retinol-binding protein 4
ROC: receiver operation characteristic
SBP: systolic blood pressure
SD: standard deviation
TC: total cholesterol
T2DM: type 2 diabetes mellitus
TG: triglycerides
TP: total protein
UA: uric acid
Urea: urea nitrogen
WC: waist circumstance
WHO: World Health Organization
WHR: waist-to-hip ratio
ZAG: zinc-α2-glycoprotein.
