# Supplementary figures and images for: Low Serum ZAG Levels Correlate With Determinants of the Metabolic Syndrome in Chinese Subjects
Source: Front Endocrinol (Lausanne). 2020 Mar 24;11:154. doi: 10.3389/fendo.2020.00154 (PMC7105689; doi:10.3389/fendo.2020.00154)

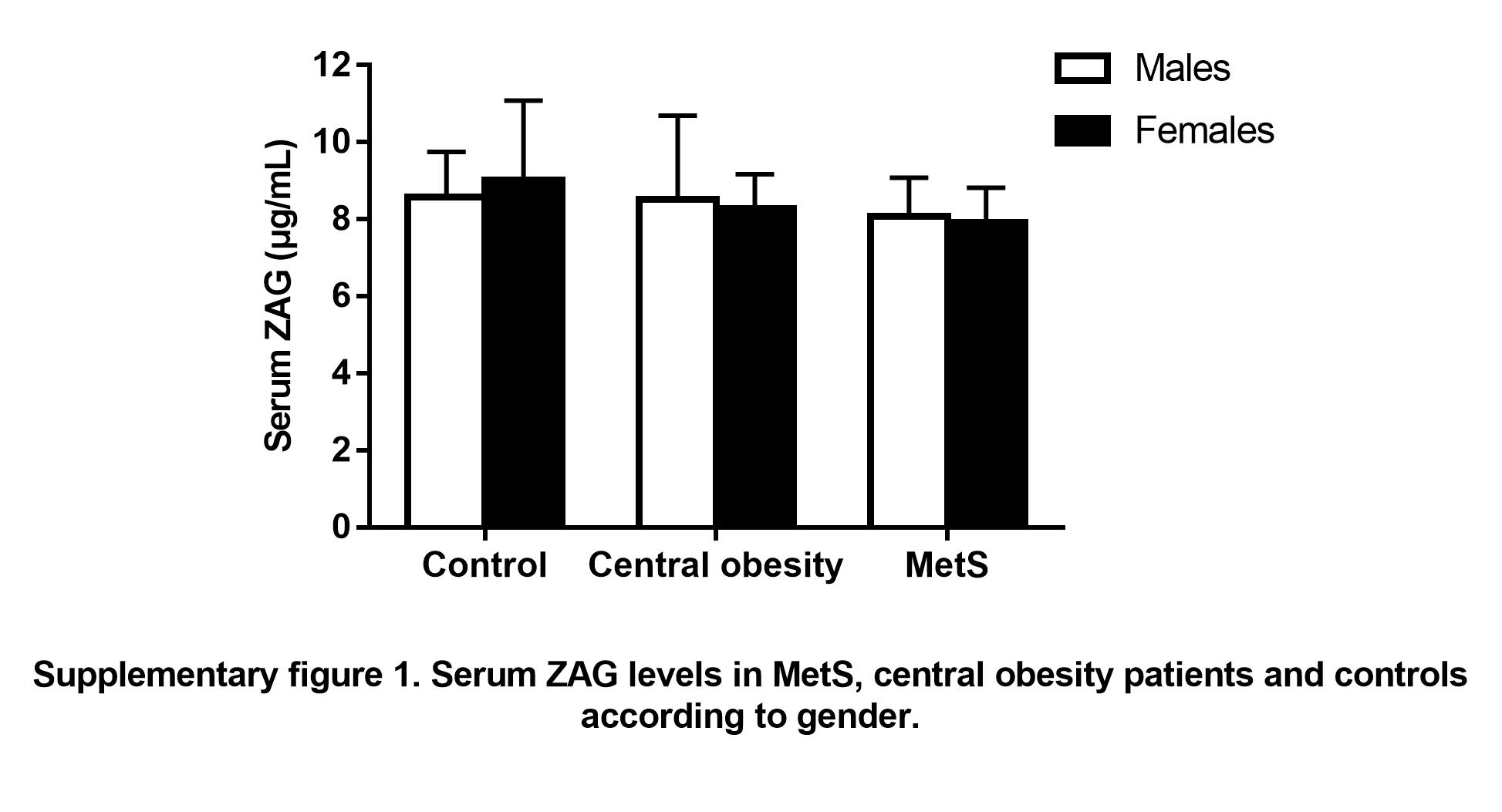

Supplement: Supplementary file 1 [file Image_1.JPEG]
